# Mammographic Breast Density Model Using Semi-Supervised Learning Reduces Inter-/Intra-Reader Variability

**DOI:** 10.3390/diagnostics13162694

**Published:** 2023-08-16

**Authors:** Alyssa T. Watanabe, Tara Retson, Junhao Wang, Richard Mantey, Chiyung Chim, Homa Karimabadi

**Affiliations:** 1 Department of Radiology, Keck School of Medicine, University of Southern California, Los Angeles, CA 90007, USA; 2 CureMetrix, Inc., San Diego, CA 92101, USArichardmantey@curemetrix.com (R.M.); homa@curemetrix.com (H.K.); 3 Department of Radiology, University of California, San Diego, CA 92093, USA

**Keywords:** automated breast density, mammography, deep learning, reader variability

## Abstract

Breast density is an important risk factor for breast cancer development; however, imager inconsistency in density reporting can lead to patient and clinician confusion. A deep learning (DL) model for mammographic density grading was examined in a retrospective multi-reader multi-case study consisting of 928 image pairs and assessed for impact on inter- and intra-reader variability and reading time. Seven readers assigned density categories to the images, then re-read the test set aided by the model after a 4-week washout. To measure intra-reader agreement, 100 image pairs were blindly double read in both sessions. Linear Cohen Kappa (κ) and Student’s *t*-test were used to assess the model and reader performance. The model achieved a κ of 0.87 (95% CI: 0.84, 0.89) for four-class density assessment and a κ of 0.91 (95% CI: 0.88, 0.93) for binary non-dense/dense assessment. Superiority tests showed significant reduction in inter-reader variability (κ improved from 0.70 to 0.88, *p* ≤ 0.001) and intra-reader variability (κ improved from 0.83 to 0.95, *p* ≤ 0.01) for four-class density, and significant reduction in inter-reader variability (κ improved from 0.77 to 0.96, *p* ≤ 0.001) and intra-reader variability (κ improved from 0.89 to 0.97, *p* ≤ 0.01) for binary non-dense/dense assessment when aided by DL. The average reader mean reading time per image pair also decreased by 30%, 0.86 s (95% CI: 0.01, 1.71), with six of seven readers having reading time reductions.

## 1. Introduction

Mammography is the cornerstone of diagnostic imaging and a widely used screening tool for breast cancer detection [1]. With advances in medical research, breast density is now recognized as an important risk factor for breast cancer development with women who have dense breasts displaying a higher risk [2]. Given its relevance, density has been incorporated into modern risk assessment tools, notably the Tyrer–Cuzick model version 8 risk assessment score [3].

One of the challenges accompanying the presence of dense breast tissue is the ability to obscure tumors, making detection more difficult on mammograms, and reducing the accuracy of the exam [4]. The Breast Imaging Reporting and Data System (BI-RADS) was developed to provide a standardized system for mammography reporting, and the BI-RADS 4th edition categorizes breast density based on the percentage of fibroglandular tissue present [5]. With continuous advancements and insights, the BI-RADS 5th edition, published in 2013, redefines the four density categories (A–D) and eliminates the percentage-based quartiles, instead, describing density distribution qualitatively based on possibly obscuring lesions [6]. In the context of regulatory mandates in the United States, qualitative BI-RADS breast density based on the BI-RADS 5th edition must be provided on mammogram reports as required by the Mammography Quality Standards Act (MQSA) regulations. Density assessments by humans based on the 5th edition are overall good, but can display intra- and inter-reader variability, with some findings of variation influenced by training and experience levels of readers [7,8]. Reader inconsistency can be a source of concern and confusion for both patients and clinicians, and can reduce reader productivity when faced with borderline cases that are difficult to classify [9]. These borderline cases not only demand additional time for evaluation but can introduce elements of uncertainty in diagnosis.

Various automated methods for density classification have been developed to address reader variability, with deep learning (DL) models being particularly promising, with the ability to determine density at the level of radiologists [10]. However, complexity arises in establishing ground truth for qualitative density categories, as this is an inherently subjective assessment and without a clear-cut ground truth. Supervised DL approaches have typically used consensus labels created by multiple readers as ground truth, but this method is susceptible to subjectivity in individual reader preferences and human bias. Therefore, to address this issue, a semi-supervised DL approach with partially labeled data (comprising some human-labeled data and a majority of unlabeled data) was used in the training of this DL model to reduce human subjectivity in ground truth labeling and enhance the consistency of the algorithm.

In this multi-reader, multi-case (MRMC) study, we assess the standalone performance of the DL model as well as the performance of seven experienced readers in mammographic density assessment with and without the aid of the DL model.

## 2. Materials and Methods

### 2.1. Model Description

**Figure 1:**
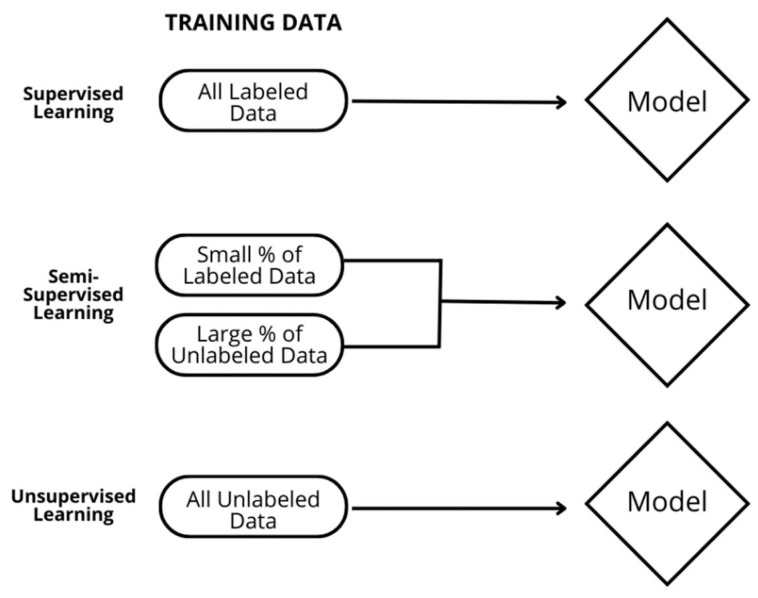
Deep learning approaches for model training. With semi-supervised deep learning, the DL model is trained with a blend of human-labeled ground truth data and a larger batch of unlabeled data. This approach is ideal in situations like breast density where the ground truth is subjective and prone to human variation and bias.

The commercially focused DL model (cmDensityTM, Curemetrix, Inc., San Diego, CA, USA) tested here was developed for automated analysis of tissue density for full-field digital mammograms (FFDM) and digital breast tomosynthesis (DBT) images based on cranio-caudal and mediolateral oblique (CC–MLO) projections. This experimental model employs a proprietary algorithm, built around an ensemble of Bayesian models [11]. Bayesian DL models allow for the estimation of uncertainty and regularize the scale of the parameters [12,13]. These models are individually trained through an iterative, semi-supervised learning approach [14,15,16] (Figure 1), during which they develop the ability to generate feature representations from paired (CC–MLO) images for the purpose of density estimation. While some details of this approach are proprietary, in general, the algorithm is trained with labeled data about breast density and is then used to predict the density of unlabeled data, referred to as “pseudo labels.” These pseudo labels are then used by the algorithm in a feedback mechanism to refine itself through Bayesian uncertainty estimates. The models are pre-trained using the imagenet-1k dataset [17]. The DL model’s outcome is determined by aggregating density estimates from all models and calculating the median value, which provides a statistically robust measure of mammographic density.

### 2.2. Reader Study Data Selection

FFDM images from 10,327 women were gathered, with exam dates ranging from April 2006 to October 2017. The data were collected from two U.S. clinical sites and are not publicly available. To ensure privacy, all data in the study, including data selection and the reader study, were anonymized through a protocol compliant with Health Insurance Portability and Accountability Act (HIPAA) regulations. The image acquisition sites approved the use of the data for algorithm development and retrospective testing, making the study exempt from Institutional Review Board (IRB) evaluation. The mammograms were filtered based on the following exclusion criteria:

- Exams with less than 4 views;
- Images with non-standard images (where standard is defined as CC and MLO);
- Images with implants (implant-displaced images were not excluded);
- Mastectomy;
- Images without corresponding ipsilateral CC or MLO view.

**Figure 2:**
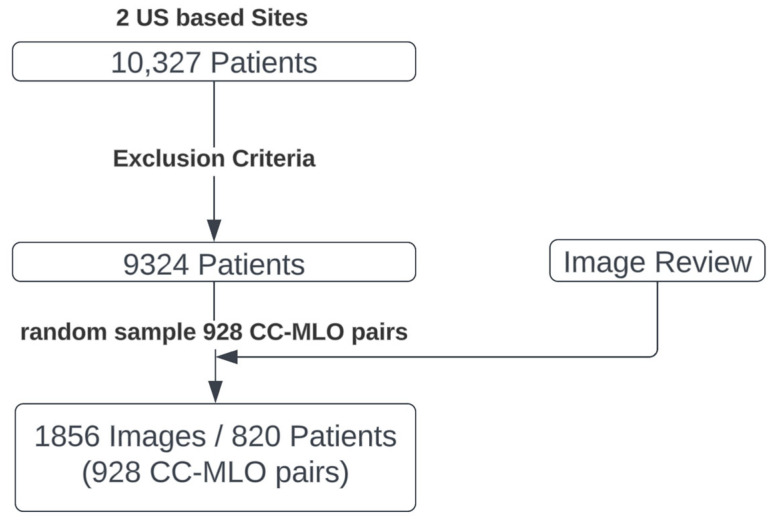
Selection process for the reader study test set. A pool of 10,327 patients with mammograms was collected. After exclusions, random sampling, and manual review, the final test set is composed of 928 cranio-caudal and mediolateral oblique mammogram image pairs (CC–MLO pairs).

There were mammograms from 9324 women after exclusion. The final test set consists of a random selection of 928 CC–MLO image pairs, representing 820 women (ages 18 to 93, median of 57 years) (Figure 2). The final test set images were acquired using two equipment vendors (Hologic, Inc., Marlborough, MA and GE Healthcare Technologies, Inc., Chicago, IL, USA). The images were stored in Digital Imaging and Communications in Medicine (DICOM) format and varied in pixel resolution from 2294 × 1914 to 6718 × 5386 pixels.

The age distribution in years of the women in the final test set was as follows: 2% (20/928) less than 40 years old, 26% (240/928) 40 to 49, 29% (267/928) 50 to 59, 24% (223/928) 60 to 69, 14% (126/928) 70 to 79, and 6% (52/928) over 80 years old. Out of the 928 CC–MLO pairs, 51% (476/928) were acquired from the left breast, and 49% (452/928) were from the right breast.

### 2.3. Reader Study Design

A 2-session MRMC reader study was conducted to evaluate the effectiveness of the DL model in aiding readers in density categorization. The reading times of the readers’ assessments were also measured in both sessions.

During each session, the readers were tasked with evaluating the density of each breast in the test set, following the 4 class (A–D) definitions of ACR BI-RADS 5th Edition where Category A = mostly fatty, B = scattered densities, C = heterogeneously dense, and D = extremely dense [5]. Each reader examined all 928 image pairs in the test set, in addition to 100 cases that were randomly selected for double reading to assess intra-reader consistency. This resulted in each reader ultimately assessing 1028 image pairs. The 100 double-read pairs were strategically interspersed among the original pairs, ensuring they were not displayed consecutively. In the first session, the readers independently assessed the 1028 image pairs without the aid of the DL model, and this is referred to as the “unaided” quantifications. This was conducted to establish a performance baseline and determine inter- and intra-reader variability. The DL model was also used to categorize the test set of 928 image pairs, and its performance was compared to that of the independent assessments from the readers.

After a 4-week washout period, the readers were presented with the same 1028 image pairs, with the same 100 pairs again presented for double reading. This time, the DL model results were presented concurrently, and the readers were asked to indicate whether they agreed with the density assessment provided by the DL model. This was referred to as the “aided” session, as readers had access to the DL model information. If the readers did not agree with the model, they assigned the density category of their choice. Various studies have demonstrated breast density grading is prone to subjectivity unlike cancer detection where the “label” can be verified through biopsy. In the absence of an objective ground-truth, the goal of this second session was to evaluate changes in reader inter- and intra-reader variability and reading times when evaluating images independently and when evaluating images aided with information from the DL model.

### 2.4. Reader Qualification

All seven readers participating in the MRMC study are MQSA qualified and American Board of Radiology certified experienced radiologists (Table 1). The majority (five/seven) of the readers interpret breast imaging as more than 75% of their total clinical workload and have more than 10 years of clinical experience. Two of the readers had 6 and 9 years of clinical experience. All readers interpret at least 500 mammograms per year, and one interprets over 10,000 annually.

### 2.5. Statistical Analysis

Intra-reader variability was assessed by comparing each reader’s density evaluations of the double-read 100 CC–MLO image pairs in both unaided and model-aided sessions. This comparison was made using pairwise Linear Cohen kappa (κ) [18], then averaged across all readers. For inter-reader variability, κ was used as well, but in this case, it was computed by comparing each reader’s assessments against those of other readers in both the unaided and aided sessions of 928 CC–MLO pairs.

The κ ranges from −1 to 1 where negative κ represents disagreements. The following thresholds were used for assessing the level of agreement: k between 0.01 and 0.20 = slight, between 0.21 and 0.40 = fair, between 0.41 and 0.60 = moderate, between 0.61 and 0.80 = substantial, and between 0.81 and 1.00 = almost perfect [18]. Standalone performance of the DL model was assessed by comparing the DL model breast density score against the median of the reader scores from the unaided sessions for both 4-class (Category A–D) and binary (dense, non-dense) classification. Dense is defined as Category C or D and non-dense is defined as Category A or B, per BI-RADS 5th edition guidelines. The corresponding *p*-values, calculated parametrically through one sample *t*-test, and 95% confidence intervals (CI) are reported. Performance measures for binary classification such as accuracy, F1, precision, recall, and balanced accuracy were also calculated, and are presented in a Supplementary Table S1.

Table 2 summarizes all the possible reader agreements in this MRMC study. The sessions of this MRMC study are either unaided or aided. All pairwise reader agreements are denoted either by Q and P and are computed using κ. Qii represents the agreement of reader i’s density grade assignment against themselves in the 100 replicated reads, and Pij represents the agreement of reader i’s density grade assignment against reader j’s. Superscript A and B represent whether the reader is unaided or aided by the DL model, respectively. Metrics are defined in the following equation which will be used for hypothesis testing.

- Inter-reader agreement for the unaided session is the average reader pairwise κ when not aided by the DL model:

$${µ}_{P}^{AA}=\frac{1}{21}\sum_{1\;\;\leq \;i\;<\;j\;\leq \;7}^{}P_{ij}^{A}$$

- Inter-reader agreement for the aided session is the average reader pairwise κ when aided by the DL model:

$${µ}_{P}^{BB}=\frac{1}{21}\sum_{1\;\leq \;i\;<\;j\;\leq \;7}^{}P_{ij}^{B}$$

- Intra-reader agreement for the unaided session is the average reader to reader themselves κ when not aided by the DL model:

$${µ}_{Q}^{AA}=\frac{1}{7}\sum_{i=1}^{7}Q_{ii}^{A}$$

- Intra-reader agreement for the aided session is the average reader to reader themselves κ when aided by the DL model:

$${µ}_{Q}^{BB}=\frac{1}{7}\sum_{i=1}^{7}Q_{ii}^{B}$$

#### 2.5.1. DL Model Standalone Performance Testing

The objective of this endpoint is to demonstrate the accuracy and consistency of the DL model in predicting the ACR BI-RADS 5th Edition breast density assessment from mammogram image pairs, as compared to the median density determined by readers for a given pair of CC–MLO images. In cases where consensus among readers cannot be reached, the median is used so that no testing data are excluded. κ was employed to evaluate the agreement between the median density grade assessment of the readers and the predictions made by the DL model in both the four-class and binary breast density assignments.

#### 2.5.2. Inter-Reader Variability Testing

The inter-reader variability endpoint is to determine whether readers’ inter-reader agreement is superior when they assess breast density grade in the aided session compared to when they assess breast density grade unaided. The samples for the test are the 928 CC–MLO pairs without the 100 double reads. Statistical testing is performed through single tail Student’s *t*-test. The corresponding *p*-value and 95% confidence interval are reported.

**H1.** 
$\begin{array}{l} \mathrm{Null}\;\mathrm{Hypothesis}:\;{µ}_{P}^{BB}-{µ}_{P}^{AA}=0\\ \mathrm{Alternative}\;\mathrm{Hypothesis}:\;{µ}_{P}^{BB}-{µ}_{P}^{AA}>0 \end{array}$

#### 2.5.3. Intra-Reader Variability Testing

The intra-reader variability endpoint is tested on the randomly selected 100 cases with a repeated reading. This endpoint is to determine whether readers’ intra-reader agreement is superior when they assess breast density grade with the aid of the DL model compared to when they assess breast density grade unaided. Statistical testing is performed through a single tail Student’s *t*-test. The corresponding *p*-value and 95% confidence interval are reported.

**H2.** 
$\begin{array}{l} \mathrm{Null}\;\mathrm{Hypothesis}:\;{µ}_{Q}^{BB}-{µ}_{Q}^{AA}=0\\ \mathrm{Alternative}\;\mathrm{Hypothesis}:\;{µ}_{Q}^{BB}-{µ}_{Q}^{AA}>0 \end{array}$

#### 2.5.4. Reading Time Testing

Reading time analysis was an exploratory endpoint of this MRMC study. The amount of time it took a reader to render a density grade assessment was recorded for both aided and unaided sessions in the unit of seconds. One sample *t*-test was used to compare each individual reader’s and the readers’ average time between aided and unaided settings.

## 3. Results

### 3.1. DL Model Standalone Performance Testing

**Figure 3:**
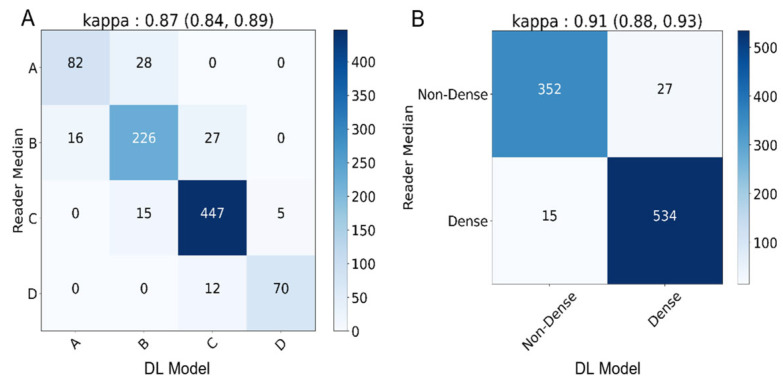
Deep learning (DL) model’s agreement compared to readers’ median consensus. (**A**) Four classes (A–D) confusion matrix of cmDensity vs. reader median assessments with a κ of 0.87. (**B**) Binary class (dense/non-dense) confusion matrix of the DL model vs. reader median assessments with κ of 0.88.

The DL model achieved an almost perfect level of κ for both the four-class (κ = 0.87, 95% CI: 0.84, 0.89) and binary (κ = 0.91, 95% CI: 0.88, 0.93) density assessments. The DL model assigned categories as follows: 10.5% A (92/928), 28.9% B (269/928), 51.1% C (486/928), and 8.1% D (75/928). In total, there are 11% (103/928) discordant image pairs when comparing against the readers’ consensus for four-class density assessment. For binary density classification, there are 4.5% (42/928) discordant assessments when comparing the DL model to the readers’ consensus. The discordant image pairs were off by one category only (such as B to C), and there were no 2 or 3 category discordances (such as A to D or vice versa). Detailed confusion matrices are presented in Figure 3. Additional measures of classification performance including precision, recall, and F1-score are presented in Supplementary Table S1.

### 3.2. Inter-/Intra-Reader Variability Testing

**Figure 4:**
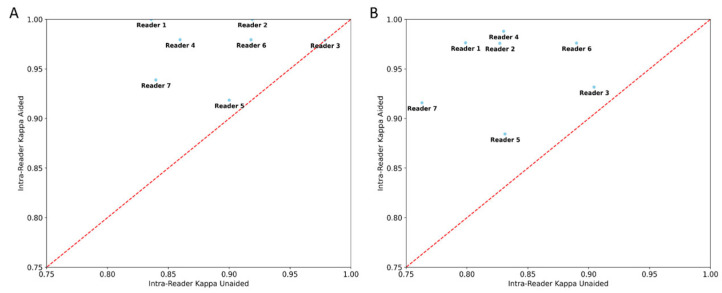
Scatter plot of reader intra-reader variability point estimates of aided vs. unaided results. Figure (**A**) scatter plot for 4-class density (A–D) assessments and (**B**) binary density (dense/non-dense) assessments. All readers had improvement in their intra-reader variability in the aided session with use of the deep learning model.

There was significant reduction (*p* < 0.05) in both inter- and intra-reader variability in the aided session. The point estimate of the average inter-reader κ improved from 0.698 to 0.882 for four-class and 0.773 to 0.956 for binary density assessment. The point estimate of the average intra-reader κ improved from 0.834 to 0.950 and 0.892 to 0.971, respectively, for the four-class and binary density assessment (Table 3). The point estimates of each reader’s intra-reader k improved in the aided session for all seven readers in both the four-class and binary assessments (Figure 4). The significant *p*-value indicates it is highly unlikely that the improvements of inter- and intra-reader variability is due to randomness.

### 3.3. Reading Time Testing

**Figure 5:**
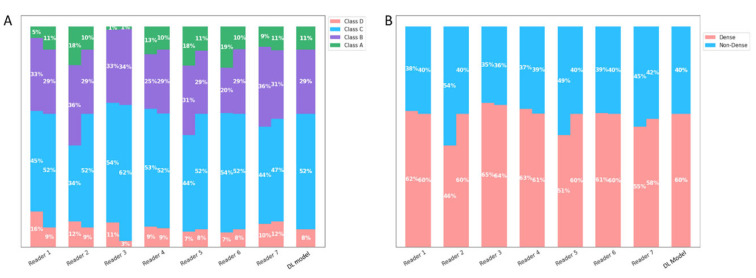
Density assessments with and without the deep learning model’s aid. This figure presents a side-by-side comparison of readers’ density assessments, distinguishing between those made without assistance and those made with the support of a deep learning model. (**A**) Represents assessments based on a 4-class density categorization (A–D). For each density class, two bars are displayed: The left bar corresponds to the unaided assessments. The right bar shows the assessments when aided by the deep learning model. (**B**) Represents assessments based on the binary density classifications: ‘dense’ and ‘non-dense’. Similar to Panel (**A**), two bars represent each classification: The left bar indicates the unaided assessments. The right bar illustrates the aided assessments. Across both panels, a key observation is the reduced inter-reader variability in the assessments when readers are aided by deep learning model.

In the aided session, the average reader mean reading time per image pair significantly decreased by 30%, 0.86 s (95% CI: 0.01, 1.71). (Table 4). Five out of seven readers showed statistically significant reduction in reading time (*p* < 0.05) with the aid of the DL model with time reductions ranging from 14% to 63% in that group. These five readers had the highest concordance with the model, ranging from 97.1% or more of the test set. Reader 7 had a 92.6% concordance with the DL model assessments in the aided session with a reading time reduction that was significant at an alpha level of 10% but not 5%. One reader had a statistically significant 24% increase in reading time. This reader disagreed with the DL model on 18.75% (174/928) of the image pairs, which was the largest disagreement among all the readers (Figure 5). However, only 21.3% (37/174) of the discordant assessments for this reader resulted in a change in the binary density grade, indicating that the impact on the classification of binary breast density, a key factor in breast cancer risk and detection, was limited in a majority of the cases.

## 4. Discussion

Breast density is a critical risk factor for the development of breast cancer, and dense breast tissue has the potential to obscure tumors, leading to complications in their detection via mammograms. This issue can, in turn, compromise the accuracy of these tests [19]. To provide women with vital information about their health, laws in several jurisdictions even mandate that women be notified if they have dense breasts, highlighting the significance of binary density grading (dense vs. non-dense) [20]. Although the BI-RADS 5th edition sought to help streamline density reporting, there is still a subjective component to density determination [7,21]. A noteworthy, large multi-center study from the National Cancer Institute involving 30 radiology facilities showed that there is an enduring and substantial reader variability for density assessments. While an average of 36.9% of mammograms in this study were rated as dense, individual reader assessments displayed marked variability, with dense categorization ranging from 6.3% to 84.5%. Furthermore, discordant categorization of dense versus non-dense was observed in 17.2% of cases [22]. These findings underscore the subjective nature of this evaluation, and illuminate the need for achieving more consistent assessments, as uniformity in density assessments has direct and tangible impacts on clinical decision making.

With a κ of 0.87 in the four-class and 0.91 in the binary assessments, the DL model shows not only high accuracy but is in the range of almost perfect agreement with reader median assessments of breast density. This study also showed significant improvement in both readers’ inter- and intra-reader variability with the aid of the DL model and reduced reader assessment time for the majority of the readers.

Various quantitative methods for density classification have been developed to address reader variability. Volumetric-based density products such as Quantra and Volpara offer continuous density estimates, but they depend on pre-determined thresholds to categorize their density measurements, which may not effectively apply to BI-RADS 5th edition definitions in all cases. Volpara and Quantra have demonstrated only moderate agreement with readers. In a retrospective study involving 1185 images, Quantra achieved a κ ranging from 0.54 to 0.61, while Volpara achieved a κ ranging from 0.32 to 0.43 [23]. A larger retrospective study with 1911 patients yielded a κ of 0.46 (95% CI: 0.44, 0.47) for Quantra and 0.57 (95% CI: 0.55, 0.59) for Volpara, indicating moderate alignment with radiologists’ assessments [24].

Another approach to quantitative assessment of breast density has been the use of deep learning. Supervised deep learning methods for interpreting mammograms often rely on consensus interpretations of several radiologists as the ground truth. However, given the inherent subjectivity in assessing tissue density, different radiologist groups could lead to varied consensus, potentially capping model performance. Studies employing this approach reported a κ of 0.78 (95% CI: 0.73, 0.82) and a lower κ of 0.67 in a larger dataset of 3649 mammograms [25,26]. In contrast, this DL model achieved a κ of 0.87 (95% CI: 0.84, 0.89), higher than the supervised model counterpart, which suggests the high effectiveness of application of semi-supervised deep learning approach in density classification.

Binary density classification carries significant implications for patients, impacting not only their notification status (as only patients with dense tissue receive direct notification regarding their tissue density and potential candidacy for supplemental screening tests) but also their Tyrer–Cuzick breast cancer risk assessment scores. Women who are classified as high risk may be eligible for genetic testing and/or supplemental screening with ultrasound and/or MRI. The consistency of the DL model may be a useful aid for improving patient outcomes. Our approach improved inter- and intra-reader agreements primarily by enhancing the consensus in classification of difficult, borderline image pairs. This study strongly suggests that the AI model can effectively aid in minimizing variability in density classification by radiologists. With the aid of the DL model, women with borderline mammograms may undergo less fluctuation in their reported density stratification, Tyrer–Cuzick risk score, and supplemental screening recommendations from year to year. Readers with the highest level of concordance with the DL model assessments also showed significant improvement in their reading time, suggesting a potential productivity benefit with the acceptance of the DL model.

As with any study, limitations are acknowledged, and here, the study was limited due to restricted vendor diversity and sample size. Additional studies will be needed to evaluate how this model may perform and impact reader decision-making in other clinical scenarios. The potential benefit of this model as an aid for DBT mammography is under investigation.

## 5. Conclusions

In conclusion, the semi-supervised DL model (cmDensity) shows high performance with almost perfect agreement compared with readers and also reduced inter- and intra-reader variability in breast density categorization. Use of this DL model offers a promising solution for improved qualitative mammographic density reporting, breast cancer risk assessment scoring, and reader productivity. The DL model may have future application in report template auto-population, sorting or distributing cases for batch interpretation, and retrieval of exams for MQSA audits, which may further enhance productivity.

## Figures and Tables

**Table 1:** The readers in the study are all experienced radiologists, all with 6 or more years of clinical practice in mammography interpretation.

Reader	Experience (Years) in Mammography	MQSA and ABR Certified	>75% of Time in Mammography	Average Number of Mammograms/Year
1	>20	Y	Y	2500
2	31	Y	Y	>10,000
3	9	Y	Y	5700
4	6	Y	N	560
5	11	Y	N	1700
6	>20	Y	Y	8000
7	>20	Y	Y	4500

**Table 2:** *Q^AA^*, *Q^BB^* represent intra-reader agreements measured by Linear Cohen Kappa of reader *i* for *i* ∈ {1,…, 7} in aided and unaided sessions. *P^AA^*, *P^BB^* represent inter-reader agreements measured by Linear Cohen Kappa of reader *i* and *j* for distinct *i*, *j* ∈ {1,…, 7} in aided and unaided sessions.

Reader Agreement		Unaided			
		Reader 1	Reader 2	…	Reader 7
Unaided	Reader 1	$Q_{11}^{AA}$	$P_{12}^{AA}$	…	$P_{17}^{AA}$
	Reader 2	$P_{21}^{AA}$	$Q_{22}^{AA}$	…	$P_{27}^{AA}$
	…	…	…	…	…
	Reader 7	$P_{71}^{AA}$	$P_{72}^{AA}$	…	$Q_{77}^{AA}$
**Reader Agreement**		**Aided**			
		**Reader 1**	**Reader2**	**…**	**Reader 7**
Aided	Reader 1	$Q_{11}^{BB}$	$P_{12}^{BB}$	…	$P_{17}^{BB}$
	Reader 2	$P_{21}^{BB}$	$Q_{22}^{BB}$	…	$P_{27}^{BB}$
	…	…	…	…	…
	Reader 7	$P_{71}^{BB}$	$P_{72}^{BB}$	…	$Q_{77}^{BB}$

**Table 3:** H1 represents the inter-reader variability test for 4-class (A–D) and binary (dense/non-dense) density assessments, whereas H2 represents the intra-reader variability test for 4-class (A–D) and binary (dense/non-dense) density assessments. There were significant improvements in all metrics in the aided session.

Test	Unaided	Aided	95% CI	*p*-Value
H1 4 class	0.70	0.88	(0.16, ∞)	1.71 × 10^−16^
H1 Binary	0.77	0.96	(0.16, ∞)	6.98 × 10^−18^
H2 4 class	0.83	0.95	(0.07, ∞)	1.03 × 10^−3^
H2 Binary	0.89	0.97	(0.04, ∞)	5.57 × 10^−3^

**Table 4:** The average reader time (seconds) in density assessment was reduced for the majority (6/7) of the readers with the aid of the deep learning based model. The reading time reduction was significant for 5 readers.

Reader	Unaided	Aided	% Change	95% CI	*p*-Value
Reader 1	3.60	1.72	−52%	(1.68, 2.07)	9.37 × 10^−69^
Reader 2	2.00	1.724	−14%	(0.18, 0.37)	5.94 × 10^−8^
Reader 3	1.64	2.04	24%	(−0.52, −0.28)	1.74 × 10^−10^
Reader 4	3.04	1.11	−63%	(1.59, 2.27)	4.08 × 10^−27^
Reader 5	1.92	1.48	−24%	(0.30, 0.58)	2.18 × 10^−9^
Reader 6	4.38	2.80	−36%	(1.32, 1.85)	2.03 × 10^−29^
Reader 7	3.51	3.20	−9%	(−0.03, 0.65)	7.23 × 10^−2^
Average	2.87	2.01	−30%	(0.01, 1.71)	4.9 × 10^−2^

## Data Availability

Restrictions apply to the availability of these data. Data were obtained from two U.S. clinical sites and are not publicly available at this time.

## Supplementary Materials

The following supporting information can be downloaded at: https://www.mdpi.com/article/10.3390/diagnostics13162694/s1, Table S1: Precision, recall, and F1-score are presented for the DL model for the four categories of breast density (A–D) and for binary assessment of dense versus non-dense.
