# Supplementary material for: Mammographic Breast Density Model Using Semi-Supervised Learning Reduces Inter-/Intra-Reader Variability
Source: Diagnostics (Basel). 2023 Aug 16;13(16):2694. doi: 10.3390/diagnostics13162694 (PMC10453732; doi:10.3390/diagnostics13162694)
Supplement: Supplementary file 1 [file diagnostics-13-02694-s001.zip › diagnostics-2512473-supplementary.pdf]

Table S1. Precision, recall, and F1-score are presented for the DL model for the four categories of breast density (A–D) and for binary assessment of dense versus non-dense.

Density	Precision	Recall	F-1 score	Case Number (N)
A	0.84	0.75	0.79	110
B	0.84	0.84	0.84	269
C	0.92	0.96	0.94	467
D	0.93	0.85	0.89	82
accuracy			0.89	928
macro avg	0.88	0.85	0.86	928
weighted avg	0.89	0.89	0.89	928
	Precision	Recall	F-1 score	Case Number (N)
Non-Dense	0.96	0.93	0.94	379
Dense	0.95	0.97	0.96	549
accuracy			0.95	928
macro avg	0.96	0.95	0.95	928
weighted avg	0.95	0.95	0.95	928
